# 

**Published:** 1889-05

**Authors:** 


Entered at the Post Office at Austin, Texas, as Second-Class Mail Matter.

Vol. IV. MAY, 1889. No. 11.

### DANIEL'S MEDICAL JOURNAL

Subscription, $[illegible] a Year, in Advance.—Single Copies, Twenty-five Cents.

F. E. DANIEL, M. D., Editor and Publisher, AUSTIN, TEXAS.

